# Pet dogs prefer to work alone than to engage in a challenging cooperative task with conspecifics

**DOI:** 10.7717/peerj.20609

**Published:** 2026-01-27

**Authors:** Juliana Wallner Werneck Mendes, Giulia Cimarelli, Marie Vindevogel, Ilka van Peer, Gerd Ladurner, Friederike Range

**Affiliations:** 1 Domestication Lab, Konrad Lorenz Institute of Ethology, University of Veterinary Medicine Vienna, Vienna, Austria; 2 Behavioural Ecology Group, Wageningen University and Research, Wageningen, Netherlands; 3 Université de Rennes, Rennes, France; 4 HAS University of Applied Sciences, Hertogenbosch, Netherlands; 5 University of Vienna, Vienna, Austria

**Keywords:** Cooperation, Economic games, Stag hunt game, Assurance game, Canis familiaris

## Abstract

Understanding the role of a partner is key to effective human cooperation. While we know that non-human animals extensively cooperate with each other, how well they understand the role of their partner is unclear. This has been explored using economic games, yielding mixed results. A previous study showed that dogs understand the role of their human partner in an economic game setting, adjusting their behavior according to the partner’s choices, but there are no clear results when it comes to dog conspecific cooperation. In this study, we tested pairs of pet dogs in the stag hunt game. In the typical payoff group, dogs had the option to perform a more challenging, cooperative action for a higher reward or work individually for a lower one. To test for a potential effect of motivation for the high value reward, we had a same reward group where cooperation or individual work led to the same reward. Dogs had minimal training and exposure to the contingencies of the game. Dog pairs from both groups only coordinated their choice on the cooperative action in 5% of the trials. Accordingly, we found that dogs were generally more likely to work individually, regardless of their partner’s actions and obtainable rewards. In the typical payoff group, dogs initially showed a greater tendency to cooperate during the first session, but this declined quickly, with dogs from pairs ultimately working alone. The low success on the cooperative apparatus was likely due to dogs not investing sufficient effort to find the solution by trial-and-error. This could be due to the fact that the high-quality reward was not good enough to invest that extra effort or a preference of dogs to work alone if given the choice. Overall, our results showed that dogs did not choose to cooperate with conspecifics, in contrast to their demonstrated success in interspecific contexts. We discuss how cooperation is potentially sensitive to contextual and social constraints rather than widespread.

## Introduction

Cooperation is present throughout the animal kingdom, but the mechanisms that drive it are not always the same. When a termite brings a grain of sand to build a mound, it might not be aware of the actions of the termites next to it, or of the fact that this individual’s actions are leading to the cooperative outcome of homing the entire group (Facchini et al., 2023). However, when two people hold a beam together to build a house, they can assess the need of one another and communicate how to better place it, adjusting to each other’s actions. This ability is thought to be one of the keys to flexible and effective cooperation in humans compared to other species (Tomasello et al., 2005).

One way to test if non-human animals understand the role of their partner is to use the string-pulling task, in which two individuals can pull on the ends of a rope simultaneously to obtain a food reward. If only one individual pulls, the rope becomes loose, and the food becomes inaccessible (Hirata & Fuwa, 2007). It is proposed that if animals understand they need a partner in this task, they should wait for a delayed partner to pull (Suchak et al., 2014) (although other measures are sometimes used, for instance, not pulling the rope in the absence of a partner (Bhattacharjee et al., 2024; Schwing et al., 2016)). This ability has been shown in chimpanzees (*Pan troglodytes*) (Hirata & Fuwa, 2007; Melis, Hare & Tomasello, 2006), Japanese macaques (*Macaca fuscata*) (Sigmundson et al., 2021), elephants (*Elephas maximus*) (Plotnik et al., 2011), hyenas (*Crocuta crocuta*) (Drea & Carter, 2009), peach-fronted conures (*Eupsittula aurea*) (Torres Ortiz et al., 2020), dolphins (*Tursiops truncatus*) (Jaakkola et al., 2018), and wolves (*Canis lupus*) (Marshall-Pescini et al., 2017). Grey parrots (*Psittacus erithacus*) (Péron et al., 2011), rooks (*Corvus frugilegus*) (Seed, Clayton & Emery, 2008), blue-throated macaws (*Ara glaucogularis*) (Tassin de Montaigu et al., 2020), giant otters (*Pteronura brasiliensis*), and Asian small-clawed otters (*Aonyx cinerea*) (Schmelz et al., 2017) could cooperate when released simultaneously but not in the delay condition. For some species, results are mixed: for instance, in Massen, Ritter & Bugnyar (2015), ravens *(Corvus corax*) hardly ever succeeded when they had to wait for a partner, and also pulled a rope in the apparatus even when they were alone, suggesting they did not understand the role of the partner (Massen, Ritter & Bugnyar, 2015); while in Asakawa-Haas and collaborators’ study (2016), ravens also had a low success rate when they had to wait for a partner, but their likelihood of waiting increased with experience, and by the end of the experiment all but one bird managed to wait at least once (Asakawa-Haas et al., 2016). Keas *(Nestor notabilis)* still pulled the rope when a (human) partner was present but the partner was not pulling, suggesting they did not understand the need of the partner or the mechanisms of the apparatus (Schwing et al., 2016). In a follow-up study, keas were trained to pay attention to the handling of the string by the human partner, which led to an increase in their cooperation both with simultaneous release and with a delayed release of the partner, but only when the rope was long; when the rope was short, requiring more coordination, they were not successful in the delay trials (Schwing et al., 2020). Moreover, when the delay was ecologically relevant (a kea had to wait for a partner that was busy), they were able to wait twice as long as in the classical delay. In a different study with a different cohort of keas, they were able to wait for their partner up to 65 s after training (Heaney, Gray & Taylor, 2017). These studies highlight that, when coordinating, animals might take different aspects into account we are not aware of, that learning and training heavily affects their performance, and that some species cannot cooperate in the delay condition, suggesting a lack of understanding the partner, or alternatively, our inability to capture that behavior with experimental set-ups.

The importance of taking socio-ecological aspects into account when investigating cooperation was further illustrated by studies with wolves and dogs (*Canis familiaris*). While both species have been shown to effectively cooperate with a human partner (Range et al., 2019), and dogs have been shown to have at least a rudimentary understanding of a human partner in a cooperative task (Martínez et al., 2023), dogs do not seem to be as cooperative with conspecifics. For instance, in the string-pulling task, pack-living dogs failed to coordinate their actions and obtain the reward (Marshall-Pescini et al., 2017). The authors discuss that tolerance may have played a key role in their poor performance, as dogs avoided interacting with the apparatus simultaneously and rather alternated the actions of pulling the string. Re-testing them with a task-experienced partner did increase the dogs’ success in the task, but it remained low, at an average of 19% success (Marshall-Pescini, Basin & Range, 2018). In another string-pulling task where dogs were from the same household and tested in the presence of the owner, likely reducing tolerance issues, dogs succeeded after multiple training steps (Ostojić & Clayton, 2014). In a study where a pair of familiar dogs could coordinate their approach to openings on a fence and obtain food, they were successful in 90% of the trials, and there was no influence of whether the reward was sharable or not; however, dogs did not monitor one another and seemed to be acting independently to solve the task (Bräuer et al., 2013). In a follow-up study using the same paradigm, dogs were as successful as wolves in coordinating to obtain the reward (Bräuer, Stenglein & Amici, 2020). These examples show it is still unclear whether dogs are less cooperative towards their conspecifics or if our tests are not appropriate to capture their cooperative behavior. While it is possible that domestication relaxed selective pressure for within-species cooperation (Range & Marshall-Pescini, 2022; Marshall-Pescini et al., 2017), free-ranging dogs typically live in groups and defend their territory cooperatively (Pal, 2015; Bonanni et al., 2011). Understanding the selective pressures that led to cooperation (or lack thereof) in dogs can also help us elucidate the evolution of cooperation in humans, as dogs and humans have shared similar environmental pressures and are thought to represent a case of convergent evolution (Topál et al., 2009).

To further understand the proximate mechanisms behind cooperation, it is important to go beyond coordination paradigms such as the string-pulling task. One limitation of such coordination paradigms is that individuals can only choose between two options: cooperate and get a reward, or not cooperate and get nothing. Therefore, these set-ups do not enable us to investigate if an individual adjusts its action based on the partner’s decision. Consequently, we cannot observe the decision making and flexibility that entails cooperation in typical contexts. Moreover, animals can achieve successful cooperation by learning associative rules such as “if partner is close by, perform the action”. The use of economic games, derived from human experimental economics, may help us bridge that gap with non-human animals. These structured games present simple choices to participants, and the result depends on the combination of the choices. Although they lack in ecological validity, they are standardized and can be used across different species and contexts (Massen et al., 2019). In the stag hunt game, individuals can choose between two options: cooperate (stag) for a high reward or work individually (hare) leading to a lower reward. But if one partner chooses the stag option while the other selects hare, the first partner receives nothing, and the second gains a low-value reward.

In an instance of a stag hunt game, Bullinger et al. (2011) trained chimpanzees to either individually forage for a lower reward (choose hare) or to cooperatively pull on a rope to obtain a higher reward (choose stag), and then tested them in pairs to choose one option or the other. Dyads coordinated on stag 91% of the time. In a follow-up study where the value of the lower reward was higher, they were less likely to leave it for the cooperative choice (Duguid et al., 2014), showing that motivation for the reward can affect decision-making. Brosnan et al. (2011) tested capuchin monkeys, chimpanzees, and humans in the stag hunt game, but using tokens that could be exchanged for rewards. Humans were more successful in cooperating for the high-value reward than chimpanzees, and chimpanzees were more successful than capuchin monkeys. Species seemed to use different strategies, with some humans and some chimpanzees seemingly predicting their partner’s choices while capuchins and some chimpanzees were relying on visually matching their partners (Brosnan et al., 2011). Martínez et al. (2024) tested dogs living in the same household in this paradigm using an adaptation of the string-pulling task, in which dogs could interact with the typical apparatus cooperatively and obtain a high-value reward (choose stag), or pull the rope of an individual apparatus to obtain a low-value reward (choose hare) (Martínez et al., 2024). Seven of eleven dyads coordinated on stag in the first session, but when the position of the tables was switched in the second session, four of the seven dyads retained their preference, indicating that only some dogs managed to coordinate with their partners and adjust to their choices, while the others chose based on a side bias.

In most string-pulling studies, individuals are trained to perform the actions step by step. This makes it challenging to pinpoint mechanisms for cooperation and whether they understand the role of the partner. Moreover, in naturalistic settings such as group hunting, animals achieve effective cooperation through observation and trial-and-error over time (Boesch, 2002). Additionally, in game theoretical models, it is essential to design experiments where cooperation is more challenging than working alone. This approach is grounded in the theoretical premise that cooperation inherently involves higher costs compared to individual efforts (Kiss, Rosa-Garcia & Zhukova, 2020; Kümmerli et al., 2007). By making cooperative tasks more difficult, we can better simulate real-world scenarios where individuals must weigh the benefits of higher rewards against the increased effort and complexity required for collaboration. Taking that into account, Wallner Werneck Mendes et al. (2024) adapted Martínez et al.’s (2024) paradigm to incorporate a minimum training strategy and a challenging cooperative alternative, in addition to controlling for side bias and stimulus enhancement (Wallner Werneck Mendes et al., 2024). They used a rotating platform with the stag and hare apparatus positioned on top. The platform could be rotated to 18 different positions before every trial, therefore the options were not consistently in one place and dogs had to actually consider the location of each option in each trial. Moreover, one of the actions was the typical rope, and the other was a drawer that had to be pulled with the paw, controlling for stimulus enhancement. Dogs were tested with their owners, who would always cooperate, never cooperate, or act randomly in different conditions. Dogs in the experimental group, who could obtain the high-value reward only when cooperating, were more likely to cooperate with their owners than dogs in a second group where the high-value reward could also be obtained individually. The results showed that dogs did, to some extent, understand the role of their partners and the consequences of their actions by acting to maximize their gains. However, since humans coordinated the timing of the cooperative action, it was not necessarily more costly in terms of energy needed to solve the task than the non-cooperative side.

Here, our aim was to test the hypothesis that dogs invest effort to learn the consequences of a conspecific’s action on their own success in a cooperative task. We controlled for side bias and stimulus enhancement using the same methods as Wallner Werneck Mendes et al. (2024). Similarly to the previously described study, we had one group with the typical stag hunt payoff matrix (henceforth “typical payoff”), and another group where the high-value reward could be obtained on both the hare and stag side (henceforth “same reward”). The rationale for the two groups was to investigate whether dogs were more likely to pay attention and learn the role of the partner when a partner was needed to obtain a better reward, in addition to control for possibility that dogs cooperate for any intrinsic motivation rather than the food reward. We selected dogs from the same household and whose owners reported to present no food aggression, as to reduce tolerance issues, similarly as in Ostojić & Clayton (2014). The stag side was more challenging to solve in terms of effort needed to invest but also resulted in a higher reward (for the typical payoff group). We predicted that (1) dyads in the typical payoff would invest effort to coordinate more on stag, where they could obtain the high-value reward. Dogs in the same payoff group on the other hand, should coordinate on hare since there is less effort, less risk, and the same reward. However, if dogs have an intrinsic motivation to cooperate, we expect similar cooperation levels in the two groups. (2) Dogs in the typical payoff group should be more likely to adjust their choice to the choice of the partner than dogs in the same reward group, *i.e*., choose the same option that their partner chose.

## Materials and Methods

### Ethical note

All methods were performed in accordance with the relevant guidelines and regulations. All procedures used in the present study were approved by the institutional Ethics and Animal Welfare Committee at the University of Veterinary Medicine Vienna, in accordance with Good Scientific Practice guidelines and national legislation (protocol number ETK-165/10/2021). Additionally, the study followed the PREPARE criteria (Smith et al., 2018) and is reported in accordance with the ARRIVE guidelines (Sert et al., 2020). Written informed consent was obtained from the owners for their participation with their animals in the study and for publication of images and data included in this article. No adverse effects were expected. Yet, the experiment could be terminated at any point in case the dog owner wished.

### Subjects

We recruited dogs by contacting dog owners through the Clever Dog Lab (Vienna, Austria) database; participation was voluntary. Owners reported no food aggression between dogs. We tested 23 dog pairs between one and 12 years of age, comparable to similar studies (Martínez et al., 2024; Wallner Werneck Mendes et al., 2024). Pairs tested together had been living with their owners and with each other for at least one year. Dogs were from different breeds or mixed breeds (see Supplemental Material). Eleven pairs were assigned to the typical payoff group and twelve to the same reward group.

### Procedure

**Figure 1:**
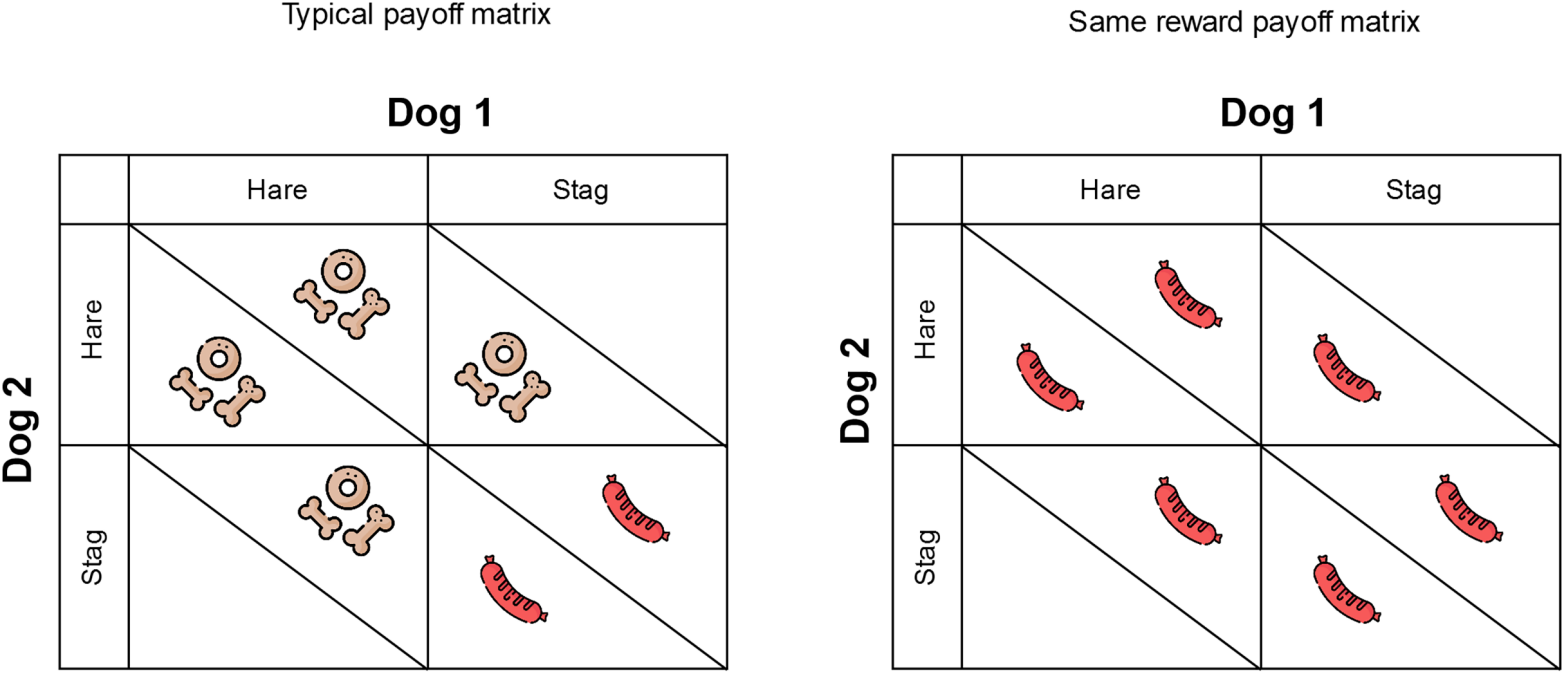
Payoff matrix for the typical payoff and same reward group. Dog treats represent low-value reward, and sausage represents high-value reward.

Based on the procedure of Wallner Werneck Mendes et al. (2024), we used the economic game stag hunt, where two individuals choose between the options stag or hare. If either participant selects “hare,” that individual earns a low-value reward. However, if both dogs cooperate by choosing “stag,” they can each received a high-value reward. In cases where one dog chooses “stag” (cooperating) and the other chooses “hare” (defecting), the cooperator receives nothing, while the defector obtains a low-value reward. In our setup, “stag” corresponded to a high-value reward (HVR) attainable only through mutual cooperation, whereas “hare” involved an apparatus that each dog could solve independently, resulting in a low-value reward (LVR). In the same reward group, the HVR could be obtained by choosing either hare alone or stag cooperatively (see Fig. 1). We used a food preference test (see Supplemental Materials for details) to determine what food was the HVR and which was the LVR. If one of the dogs of the dyad did not form a preference, the dyad was included in the same reward group. For the remaining of the allocation of dogs in the same reward group, we used the “Research Randomizer” web application (https://www.randomizer.org/).

### Apparatus

**Figure 2:**
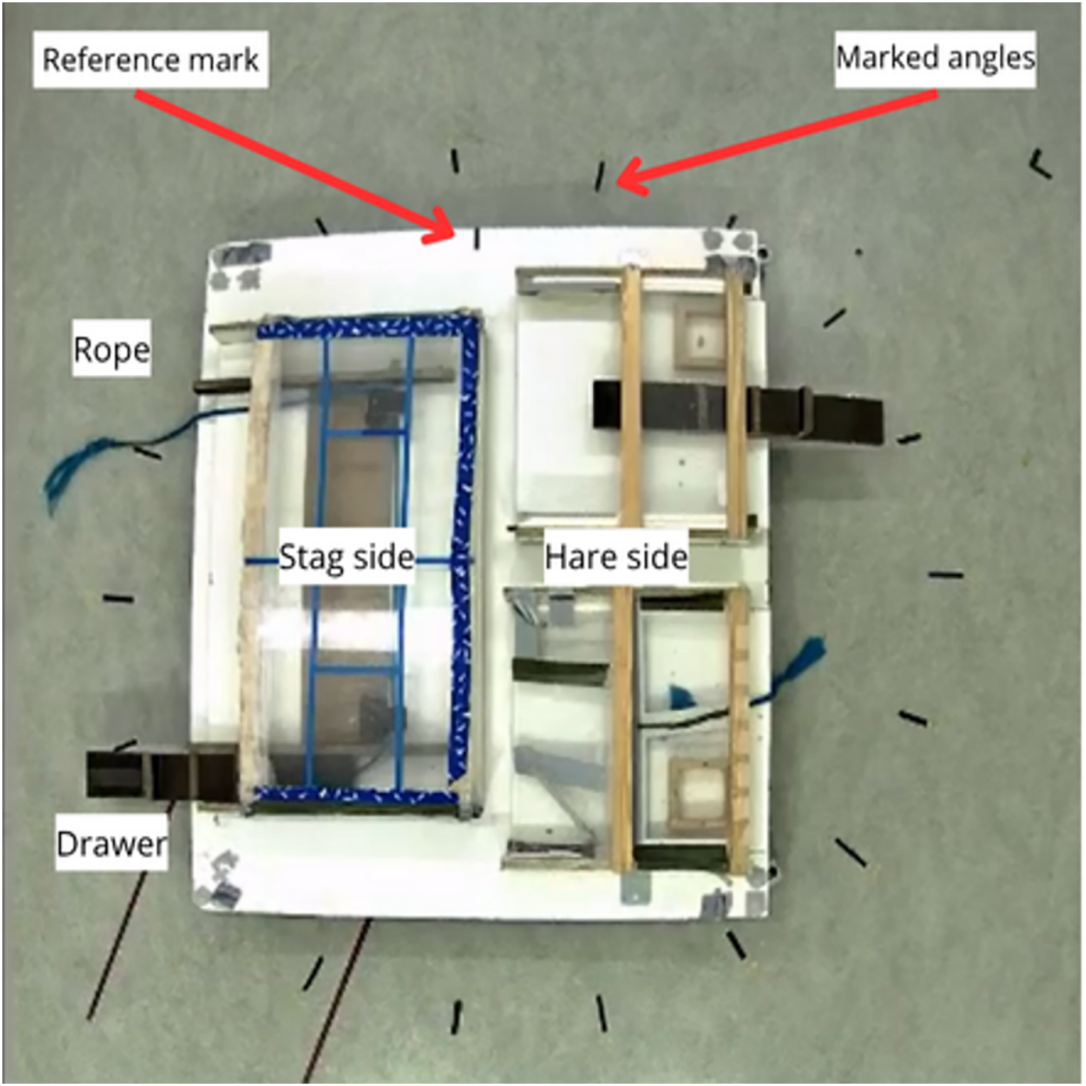
Experimental apparatus. The platform could be rotated so that the black marking (centre top) would align with one of the 18 markings on the floor. The cooperative table (stag side) can be seen on the left and the two individual tables (hare side) on the right side of the picture.

The experiment happened in a quiet room of 3.3 m by 6 m. The apparatus consisted of a platform (1.8 m × 1.5 m) that could be rotated to 18 different angles marked with tape on the floor. The cooperative table (1 m × 60 cm) was marked with blue tape as to be visually distinct from the individual tables (50 cm × 60 cm each) across from it. The cooperative table was covered by transparent plexiglass that made the food inaccessible. It had a rope on one end, and a drawer in the other. If both were pulled at the same time and at similar intensities, the table slid forward, and the food became accessible. The individual tables were likewise covered with plexiglass, and one of them had a rope and the other a drawer that could be pulled to make the reward accessible (Fig. 2). Therefore, performing the right action was enough to obtain the food on the individual side. On the cooperative side, dyads had to coordinate timing and intensity, and persist in the motion, making the reward more challenging to obtain. The apparatus was the same as in Wallner Werneck Mendes et al. (2024).

### Training

One of the dogs was trained to pull on a rope with the mouth to move a platform with a reward into reach, while the other was trained to pull a handle with the paw. We trained them on different actions to avoid that dogs would perform a certain action in the experiment based on stimulus enhancement. Which dog learned each action was consistent throughout the experiment. To minimize their familiarity with the task and the test apparatus, they were trained on a different apparatus with the same action and a different food reward. See details of training in the Supplemental Information. If one of the dogs in the dyad did not learn the task after three training sessions, the dyad was not tested. This was the case for four dyads. During the first test session, if a dog did not perform the action for five trials in a row, a training session was conducted on the test apparatus. If after this procedure the dog still did not perform an action for five trials in a row, the experimenter interrupted the experiment and verbally encouraged the dog to interact with the individual side of the apparatus three times in a row. This was the case for 16 dogs. After this procedure, all dogs performed an action during the first session of the experiment and no animal was excluded. Therefore, final sample size consisted of eleven pairs in the typical payoff group and twelve in the same reward group.

### Testing: typical payoff group

Each dyad participated in six sessions of 30 trials each. Sessions happened on different days. In each session, dogs had a break of five to 10 min halfway through. Before the start of the experiment, we randomized the position of the apparatus for each trials, using the 18 markings on the floor separated by 20 degrees each. The dog that would be released first was randomly defined using the “Research Randomizer” web application, and that was consistent throughout the experiment.

At the start of the experiment, the owner held the dogs behind an opaque partition in order for them not to see the set up. After rotating the apparatus to the right angle, the experimenter baited the tables, placing the high-value reward (HVR) on the cooperative and the low-value reward (LVR) on the individual table. The experimenter went back behind the partition and said “okay” and the name of the first dog, signaling for the owner to release them. Two seconds later, the experimenter said “okay” and the name of the second dog, and the owner released them. If the dog touched the handle of a drawer with a paw or picked up a rope with its mouth, they were considered to have made a choice. However, the choice only led to obtaining the food if the dog chose hare or if they effectively coordinated on stag with the other dog (*i.e*.,: pulling at the same time with similar intensities, so that the table slid forward and food became accessible). If the dog made a choice not leading to the access to food (*i.e*., choosing stag when the other dog already chose hare), they were called back by the experimenter to the initial position and they were not allowed to make a second choice. If one of the dogs did not make a choice within 1 min, the trial ended. The experimenter positioned the table at the next angle and placed food again where necessary, while the dogs waited behind the opaque partition held by the owner. This followed in every subsequent trial.

### Testing: same reward group

The procedure was exactly the same for the same reward group, except they had the HVR in the individual and cooperative table.

### Coding

We recorded videos using three different cameras in the experimental room from the Clever Dog Lab. We coded them on Solomon Coder beta 09.08.02 (copyright 2019 by András Péter, developed at ELTE TTK Department of Ethology, Budapest, Hungary). We report our interest variables and their definitions in Table 1. A total of 20% of the videos were coded by a second coder, and interobserver reliability was appropriate for every variable (Cohen’s kappa >0.75).

**Table 1:** Ethogram of coded behaviours, adapted from Wallner Werneck Mendes et al. (2024). All behaviours were coded for both dog 1 and dog 2. All behaviours have binary responses.

Behavior	Definition
Choice: hare	The dog touches the rope with its mouth or drawer with its paw in the individual table
Choice: stag	The dog touches the rope with its mouth or drawer with its paw in the cooperative table
Success	The dog eats the food in the chosen table
Side: right	Drawing an imaginary line exactly in the middle of the room, the dog chooses the table on the right side of the line
Side: left	Drawing an imaginary line exactly in the middle of the room, the dog chooses the table on the left side of the line
Distance: close	The dog chooses the table closest to their starting point
Distance: far	The dog chooses the table furthest to their starting point

### Statistical analysis

As the first dog to be released was not necessarily the first to make a choice, we considered “dog 1” as the first dog of the dyad to make a choice (see ethogram) on that trial. “Dog 2” was the second dog to make a choice. Consequently, the first dog to be released was always the same throughout the experiment, but which was dog 1 and dog 2 could differ. In both models we included the trials in which dogs made a choice.

Similarly to Wallner Werneck Mendes et al. (2024), we used a generalized linear mixed model (GLMM) with binomial distribution (yes/no) to estimate the effects of group and session on the likelihood of dyads coordinating on stag, (from here on, model 1). We included the interaction of group and session as the predictor. To control for a potential side or distance bias, we included chosen side and distance in the model. To account for possible differences in age of the dogs, we included age of both dogs. As proposed by Barr et al. (2013), to avoid the model being overconfident with regard to the precision of fixed effects estimates and to keep type I error rate at the nominal level of 5%, we included all theoretically identifiable random slopes. Specifically, we included the random slopes of session, chosen side, distance, and age. Before fitting the model, we z-transformed session and age to a mean of zero and a standard deviation of one, and dummy coded the categorical variables—chosen side and distance—and centred the dummy coded variables at left, and close, respectively, before we included them in the random slopes (Schielzeth, 2010). Following Forstmeier & Schielzeth (2011), as an overall test of the effect of the fixed effects and to avoid “cryptic multiple testing”, we compared the full model with a null model that was identical, except it lacked the fixed effects.

To estimate the effects of group and the choice of dog 1 on the choice of dog 2, we used a GLMM with binomial distribution (from here on, model 2). As we expected that the influence of the partner’s choice might be more pronounced in the typical payoff group and that a possible learning effect would be different according to group and partner’s choice, we included the interaction of group, partner’s choice, and session. We included age of dog 2 as a control predictor. As for the previous model, we included chosen side and distance as control predictors, and well as the random slopes of trial, chosen side, distance, and the interaction between session and partner choice. We z-transformed trial, session, and age to a mean of zero and a standard deviation of one, and dummy coded the categorical variables—chosen side and distance—and centred the dummy coded variables at left, and close, respectively. We compared the full model with a null model without the fixed effects. To simplify the model and ease the interpretation of the main effects, we removed non-significant interactions (one at a time) until we obtained a model with only main effects and significant interactions. We tested the effect of individual fixed effects by comparing the full model with reduced models lacking them one at a time (Barr et al., 2013). For these tests as well as the full-null model comparison we utilized a likelihood ratio test (Dobson & Barnett, 2002).

We performed a *post-hoc* descriptive analysis of whether dogs were making their choices simultaneously or taking turns when they coordinated. For coordination on hare, we took the latency to choose and to succeed of dog 1, and the latency to choose of dog 2. If the latency for dog 2 to choose was between the latency to choose for dog 1, and the latency to succeed for dog 1, the choice was considered simultaneous (*i.e*.,: dog 2 interacted with the apparatus between the time that dog 1 interacted with the apparatus and ate the reward, finishing the interaction with the apparatus). For coordination on stag, we considered making a choice in an interval of three seconds as simultaneous, and in a bigger interval as non-simultaneous.

## Results

In the same reward group, both dogs chose hare on 1,061 out of 1,701 trials (62%) and coordinated on stag in 93 trials (5%). In the typical payoff group, both dogs chose hare in 938 of the 1,365 trials (69%) and coordinated on stag in 72 trials (5%) (Table 2). In the same reward group, when dog 2 chose hare, he/she succeeded in obtaining the food in 95% of the trials regardless of the partner choosing hare or stag. When dyads coordinated on stag, dog 2 succeed 8% of the time. In the typical payoff group, when dog 2 chose hare, he/she succeeded in 95% of the trials if dog 1 also chose hare, and in 90% when dog 1 chose stag. When they coordinated on stag, dog 2 succeeded in 21% of the trials. In 2% of the trials in both the same reward and the typical payoff group, dog 2 succeeded in getting a reward from the stag option despite the fact that dog 1 chose hare. This was an artifact caused when the dog pulled the table hard enough and at a particular angle so that it slid forward, allowing the dog to put the snout under the Plexiglas far enough to obtain the reward (Table 2).

**Table 2:** In bold, matrix of choice of the dyads, in the same reward and typical payoff group. Below that, the rate of success of dog 2, when that choice was made. In brackets, how often dog 1 made that choice.

		Dog 2	
		Hare	Stag
**Same reward group**			
Dog 1	Hare (74%)	**Chosen: 62%**	**Chosen: 12%**
		When chosen, dog 2 succeeded: 95%	When chosen, dog 2 succeeded: 2%
	Stag (26%)	**Chosen: 20%**	**Chosen: 5%**
		When chosen, dog 2 succeeded: 95%	When chosen, dog 2 succeeded: 8%
**Typical payoff group**			
Dog 1	Hare (82%)	**Chosen: 69%**	**Chosen: 14%**
		When chosen, dog 2 succeed: 94%	When chosen, dog 2 succeeded: 2%
	Stag (18%)	**Chosen: 12%**	**Chosen: 5%**
		When chosen, dog 2 succeeded: 90%	When chosen, dog 2 succeeded: 21%

**Figure 3:**
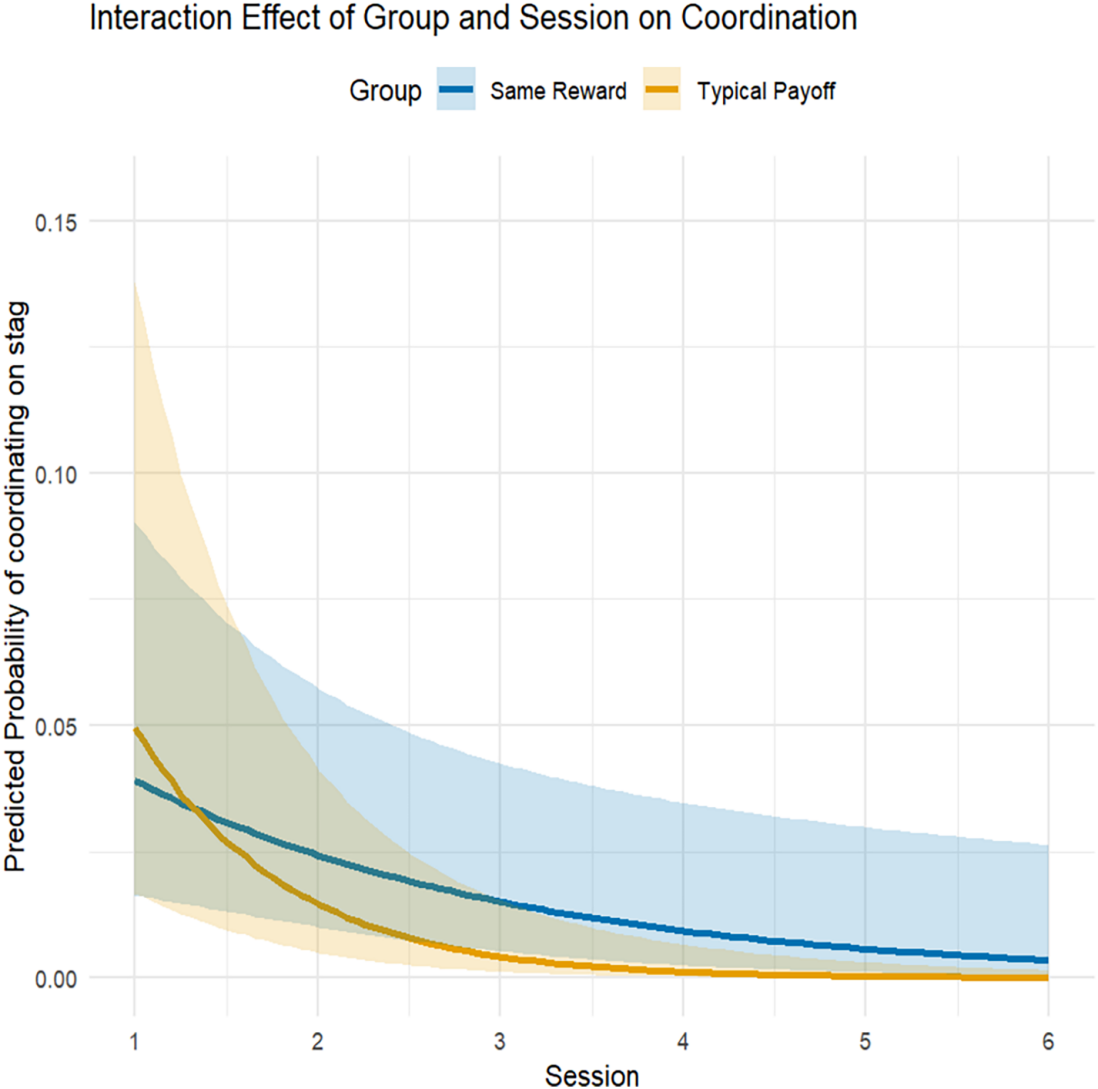
Plotted effect of session for the same reward and typical payoff group. The lines refer to the model estimates and the ribbons to the confidence interval.

For model 1 (coordination on stag: yes/no), the predictors had a significant effect on the likelihood of dyads coordinating on stag ( $\chi^2$ = 29.69, df = 3, *P* < 0.0001). The interaction of group and session had a significant effect ( $\chi^2$ = 5.36, df = 1, *P* = 0.02, Table 3) with dogs in the typical payoff group being more likely to initially coordinate on stag, but then decreasing more steeply in the following sessions (Fig. 3) compared to the same reward group. Moreover, the control predictors “distance dog 1” and “distance dog 2” were significant (Table 3): both dogs were more likely to coordinate on stag when that option was closer to them at the start of the trial.

**Table 3:** Results of model 1 (estimates, standard errors and significance tests). Side is centred on “left” and distance is centred on “close”.

Term	Estimate	SE	$\chi^2$	df	*P*
Intercept	−6.21	0.95	–	–	–
Age dog 1	−0.21	0.23	0.88	1	0.35
Age dog 2	0.33	0.25	1.90	1	0.17
Distance dog 1	–	–	7.19	2	0.03
Front (*vs* Back)	0.92	0.39	–	–	0.02
Middle (*vs* Back)	−0.83	1.74	–	–	0.63
Distance dog 2	–	–	15.25	2	0.00
Front (*vs* Back)	1.78	0.50	–	–	0.00
Middle (*vs* Back)	1.83	0.77	–	–	0.02
Side dog 1	–	–	5.02	2	0.08
Middle (*vs* Left)	−0.93	1.37	–	–	0.49
Right (*vs* Left)	0.65	0.35	–	–	0.06
Side dog 2	–	–	1.40	2	0.50
Middle (*vs* Left)	−0.12	0.59	–	–	0.83
Right (*vs* Left)	0.33	0.31	–	–	0.28
Group*Session	–	–	5.36	1	0.02
Control (at mean session)	−5	1.02	–	–	–
Experimental (at mean session)	−6.76	1.12	–	–	–

**Figure 4:**
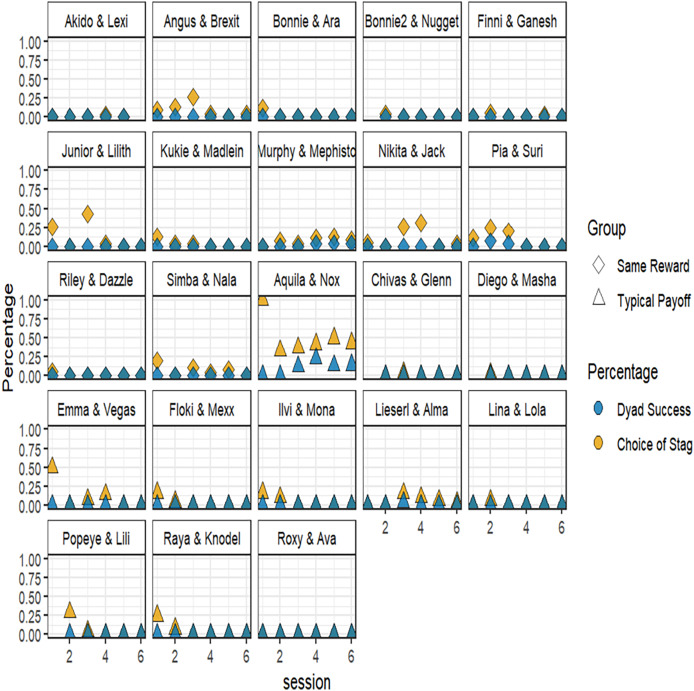
Likelihood of dyads coordinating on stag (in yellow) and likelihood of dyads succeeding when coordinating on stag (in blue).

Visual inspection of the data separated per dyad showed that one dyad (Aquila and Nox) cooperated and succeeded more consistently than all other pairs: both dogs chose stag in 44% of the trials and succeed in 16% of these trials, which was consistent across sessions (Fig. 4).

Looking more closely at dyads’ coordination on hare, we found that dogs were making their choices simultaneously in 26% of the trials, but taking turns to manipulate the apparatus in the remaining 74%. In contrast, for coordination on stag, dogs made their choices simultaneously in 49% of trials.

**Figure 5:**
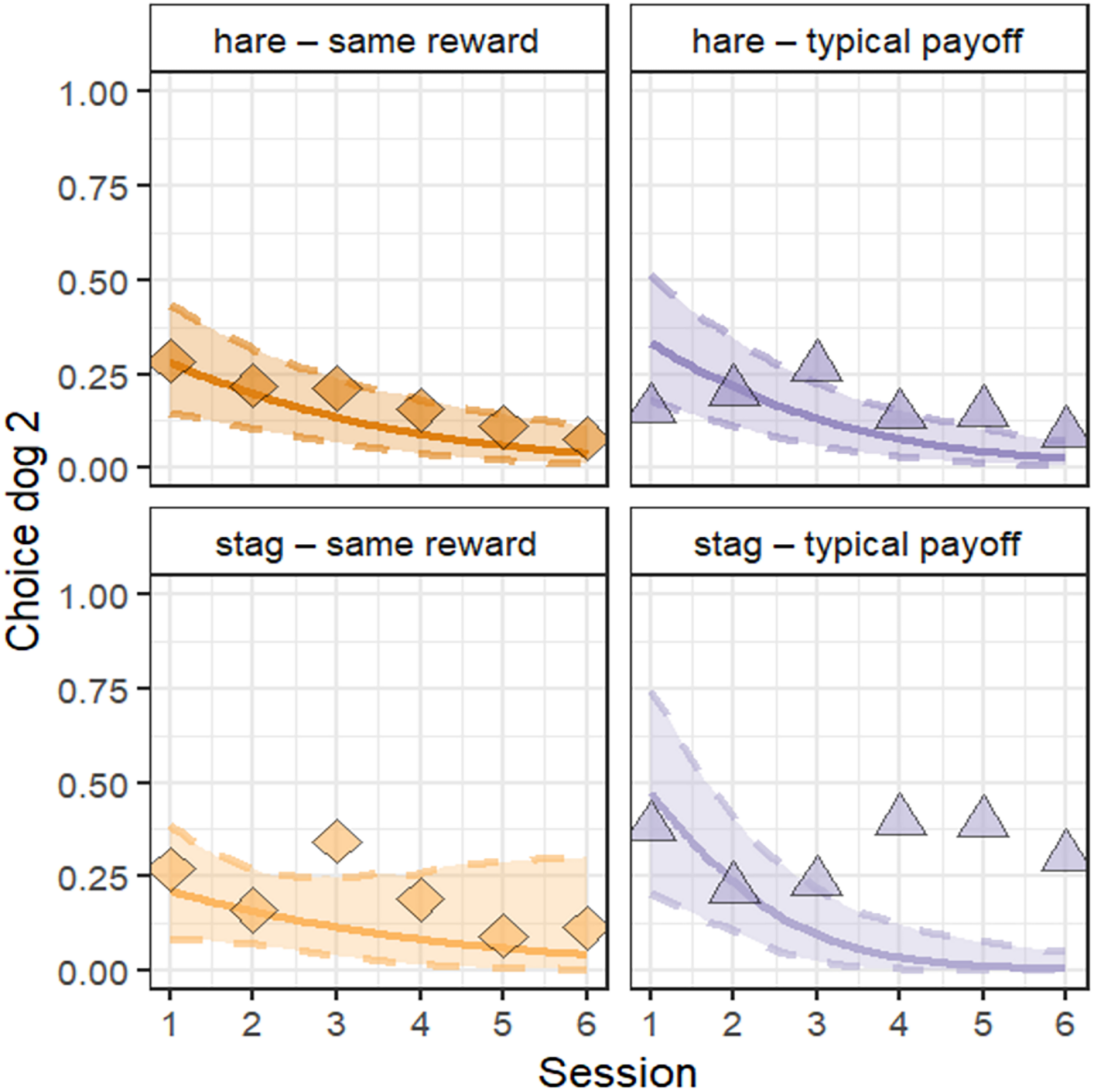
Plotted effect of session on choice of dog 2. Group and choice of dog 1, which did not have a significant effect, are displayed. The lines refer to model estimates, ribbons to the confidence intervals, and the shapes to the means from the raw data.

The full-null comparison for model 2 (choice of subject: hare/stag) revealed that the predictors (partner choice, group, and session) had a significant effect on the dogs’ choices ( $\chi^2$ = 23.25, df = 7, *P* = 0.0015). Removing non-significant interactions one at a time, only session was significant ( $\chi^2$ = 17.94, df = 1, *P* < 0.0001; Table 4). We did find not a significant interaction between partner choice, group, and session. The likelihood of dog 2 choosing stag decreased across sessions, regardless of group or choice of dog 1 (Fig. 5).

**Table 4:** Results of model 2 (estimates, standard errors and significance tests). Side is centred on “left” and distance is centred on “close”.

Term	Estimate	SE	$\chi^2$	df	*P*
Intercept	−2.43	0.41	–	–	–
**Choice dog 1**	−0.13	0.42	0.25	1	0.62
**Group**	−0.13	0.53	1.36	1	0.24
**Session**	−0.64	0.19	17.94	1	0.00
Trial	−0.15	0.08	3.70	1	0.05
Age dog 2	0.43	0.20	3.98	1	0.05
Distance dog 2:	–	–	2.38	2	0.30
Front (*vs* Back)	0.29	0.18	–	–	0.11
Middle (*vs* Back)	0.20	0.45	–	–	0.65
Side dog 2	–	–	0.86	2	0.65
Middle (*vs* Left)	0.00	0.33	–	–	1.00
Right (*vs* Left)	0.16	0.17	–	–	0.35

## Discussion

We investigated whether dogs invest effort to learn the consequences of a conspecific’s action on their own success in a cooperative task, using an adaption of the string-pulling task and the stag hunt game. At the start of the experiment, dogs in the typical payoff group were more likely to coordinate on stag compared to those in the same reward group, but the probability to do so decreased more steeply in comparison to the same reward group. Dyads from the same reward and typical payoff groups were more likely to both choose hare and seldom coordinated on stag (approximately 5%). We did not find support for the hypothesis that dogs adjusted to the choice of the partner: subjects were overall more likely to choose hare than stag, independently of the choice of the partner or group; moreover, subjects’ choice of stag decreased across sessions. These results show that dogs attempted to explore different contingencies at the beginning of the experiment, but quickly learned to obtain food on the individual side, decreasing their choice of the cooperative side. The fact that dyads in the typical payoff group were more likely to coordinate on stag, although at a small rate, does suggest that dogs were interested in the high-value reward and were attempting to obtain it. Overall, there was no consistent cooperation to obtain the high-value reward except for one dyad (Aquila and Nox), that coordinated on stag in 44% of the trials and succeeded in 16% across all sessions. Moreover, dogs in the typical payoff group were not more likely than dogs in the same reward group to adjust to their partner’s choice. These results do not support the hypothesis that dogs invest effort to learn the consequences of the actions of their partner.

Our results that dogs did not consistently cooperate contrast with findings from a similar study, where at least a subset of dogs managed to coordinate with another dog on stag (Martínez et al., 2024). This difference might be explained by varying training processes. Martínez and collaborators trained dogs specifically to pull the apparatus with a partner: after learning how to pull the individual side for a reward, they were trained with a human who pulled at the same time as the dog; then they were trained to wait for the human partner to arrive before pulling; and finally they were trained with a conspecific stooge that had been previously trained to pull the cooperative apparatus. Moreover, dogs were exposed to the testing set-up and obtained rewards from both the hare and the stag side before the experiment started. Similarly, in Bullinger et al. (2011), where dyads coordinated 91% of the time, the chimpanzees were trained in how to obtain food in hare, how to cooperatively obtain food in stag, and the consequences of each action. In contrast, the present study used minimal training and a set up that required dogs to explore the contingencies and learn how to obtain the high-value reward through a process of trial-and-error learning—a more comparable situation to cooperation would enfold in naturalistic settings. Interestingly, under these more difficult conditions, dogs were not able to cooperate with a conspecific. This illustrates the amount of experience and instruction that dogs need to achieve successful conspecific cooperation. It could be argued that the modifications in the procedure made the task too complex for dogs to figure out the contingencies, which is a common challenge when using economic games with non-human animals (Massen et al., 2019). However, a previous study testing pet dogs in the same set up (rotating table, two different actions, minimal training) with their owners, found that dogs coordinated on stag with their owners, and did so more frequently in the experimental group than in the same-reward group (Wallner Werneck Mendes et al., 2024). Indeed, humans were instructed to be predictable, and therefore it was likely easier for the dogs to adjust to their behavior. With dogs being tested spontaneously, this predictability is impossible to achieve, and we argue that it emulates naturalistic cooperative interactions such as hunting in a pack, where one individual is not necessarily acting in a specific way to facilitate the actions of the other. Moreover, the cooperative action being more challenging is an intentional and important aspect of the task, as cooperation usually is necessary when getting the reward is harder (and not possible to reach alone) and of a higher value (MacNulty et al., 2014).

There are different possible explanations for dogs’ high success in cooperating with humans but not conspecifics. Many studies have shown that dogs are highly attentive to humans (Martínez et al., 2023; Mongillo et al., 2010; Ogura et al., 2020), and in fact more attentive to humans than to conspecifics in social interactions (Törnqvist et al., 2015), which might have allowed them deduce the role of the human’, but not conspecific’s actions. Moreover, as mentioned above, since the humans were instructed to coordinate effectively once the dogs decided to coordinate on stag, making cooperation less costly than in the present study, success rate was higher than when two dogs attempted to reach a solution by trial-and-error. When it comes to conspecific cooperation, pack dogs failed in the string-pulling task by taking turns when interacting with the apparatus rather than manipulating it at the same time (Marshall-Pescini et al., 2017). Here we chose pet dogs, who had a history of being tolerant towards their partners in the food context, and we had the owner being present. In our study, when both dogs chose hare, they did so non-simultaneously in 74% of the time. When both dogs chose stag—where coordinating in time was one of the requisites to succeed—they did it simultaneously in approximately half of the trials. Therefore, avoidance was not likely the reason for failure when both chose stag. Rather than a social constraint, it is likely motivation issues, alongside dogs poor means-end connection (Osthaus, Lea & Slater, 2005), strongly influenced subjects’ performance: dog dyads tended to walk away from the cooperative side if they did not succeed with a single swift pull (as they pulled with different intensities, leading to partial movement of the table) instead of persisting on the action. This is consistent with other studies that found low persistence of dogs to try to solve tasks (Lazzaroni et al., 2020; Rao et al., 2018). While dogs’ success on the hare side was contingent only on performing the right action, the success on the stag side, as it was planned to be more challenging, depended on (1) the dog performing the right action; (2) the partner also choosing stag and performing the right action; (3) both dogs performing the right action at the same time and similar intensity. Therefore, after non-rewarded attempts on the stag side, dogs might have learned to avoid that side and obtain food elsewhere. This is consistent with the fact that, when choosing hare, subjects had a success rate of at least 90% regardless of partner’s choice in both groups. However, even when both individuals chose stag, the success rate was of 8% in the same reward group and 21% in the typical payoff group, as shown in Table 2. These low rates are comparable to the success rate of pack hunting in wild canids, for example the 20% average for wolves (Mech, Smith & MacNulty, 2015). This illustrates how obtaining food cooperatively is challenging and contingent on persistence and repetition. For instance, is Brosnan, Wilson & Beran’s (2012) study, only one out of four pairs of capuchins achieved the stag-stag outcome when they were tested in a 40 trials session per day, but all achieved it when they were tested in multiple 60 trial blocks for 2 h a day. Another explanation is that, although we chose a food reward that the dogs preferred, it is possible that the higher cost of the cooperation did not match the increase in the quality of the reward, leading dogs to repeat the cheaper hare action rather than persist in challenging one, a pattern seen in other species (Evenden & Robbins, 1984; Lee et al., 2004). In our experiment, dogs prioritized the safer, lower-reward option rather than persisting in the potential for cooperation.

The higher success rate of the typical payoff group is explained by the one dyad that succeeded above average, Aquila and Nox. Although an exception, they serve as evidence that the cooperative task is solvable. Aquila (female, 9 years old) and Nox (male, 1 year old) are border collies (as nine other dogs in our experiment, see Supplemental Information), unrelated, with basic obedience training. Aquila sporadically participated in activities such as agility, search (for leisure), and dog dancing, which was similar to other dogs participating in this experiment. As their breed, training, and daily life did not greatly differ from other dyads in the experiment, it is possible that the relationship between them might have led to the higher levels of success in the task. Friendship has been shown to be positively associated with cooperation in long-tailed macaques (Bhattacharjee et al., 2024), and keas with higher affiliation score were more successful in cooperating (Schwing et al., 2016). A study with wolves showed that the relationship between partners was a better predictor of effective cooperation than non-social factors (Dale, Marshall-Pescini & Range, 2020). For both dogs and wolves, a stronger bond promoted more sharing of food between individuals (Dale et al., 2017). Also dogs can form close relationships with each other (Cimarelli et al., 2019) and the quality of relationship affects stress buffering (Cimarelli et al., 2021). Therefore, it is likely that the quality of relationship also affects cooperation in dogs, an aspect that could be investigated in further studies.

The challenge of finding the stag-stag payoff is also illustrated by other species. For instance, in a study where individuals also had to find the payoff matrix by themselves, primates, including humans, also coordinated on stag at very low rates: one out of six capuchin pairs, five out of 25 human pairs, none of the ten pairs of chimpanzees from the MD Anderson Cancer Center, and two out of four pairs of chimpanzees from the Language Research Center, where they are more cognitively enriched and used to interactive tasks (Brosnan et al., 2011). Some chimpanzees and capuchins in Brosnan and collaborators’ study were tested in different pairs and behaved differently; for instance, two capuchins showed non-random choices with one partner but not another. For the four chimpanzees that were tested in multiple pairing, only one didn’t change behavior with different partners. This again shows that different relationships might influence cooperation. Rhesus macaques (*Macaca mulatta*) tested with a computer algorithm were, as our dogs, also making their choices without taking the choice of the partner into consideration, although, differently for the dogs in our study, they were more likely to choose stag (Parrish et al., 2014). Although a direct comparison between the studies is not possible given the different experimental set-up and the data analytical approach, the present results taken together with the primate ones, highlight that cooperative choices are not widespread, although present at a species level. This suggests that cooperation is potentially sensitive to contextual and social constraints that make it difficult to detect. Thus, it is important to investigate cooperation in a variety of species and in a variety of tasks.

## Conclusions

Overall, dogs were able to learn a solution in the stag hunt game that led to a food reward, but they were not willing to persist and cooperate with each other for a higher-value reward. Since previous research showed dogs succeed with humans in the same task, their cognitive abilities do not seem to be the limiting factor. It is nonetheless important to point out that dogs did struggle to obtain the reward even when both chose stag, leading them to settle for hare instead of persisting and eventually attaining successful cooperation. Therefore, our minimal training approach led to different results than previous studies. This shows that in previous, similar cooperation experiments, simpler associations might have led to a successful cooperative outcome, and it highlights the importance of accounting for training when investigating animals’ understanding of the role of a partner. In a theoretical review of cooperation experiments, Noë (2006) discusses that when two animals hunt simultaneously but independently, this is likely to increase their chances of succeeding, but nothing differentiates their behavior from foraging; yet, if this event occurs often enough, reinforcement of behaviors that improve coordination are possible. In most experimental paradigms testing cooperation, it is likely that animals learn the contingencies of their actions (and in some cases of their partner’s actions) through this process, and active training can facilitate animals’ learning but make our interpretation more challenging. Indeed, in studies where the coordination task was less demanding, dogs did reach a high success rate (Bräuer et al., 2013; Bräuer, Stenglein & Amici, 2020), although that does not allow to investigate whether they understand the role of the partner, as they seemed to work independently and not pay attention to each other in the task.

A limitation of our study is that in our set up it is challenging to disentangle if dogs were unable to cooperate because they did not understand that role of their partner or because they lacked a simpler skill of physically coordinating on the task (which led to some dogs needing extra exposition to the apparatus). While we present evidence that dogs prioritize working alone compared to persisting and solving a cooperative problem, it would be valuable to manipulate the value of the higher reward to test at which point dogs prefer to cooperate than to work alone, to assess their investment in learning if presented only with a cooperative version, to investigate their behavior in the absence of a partner, or to observe naturalistic multi-dog interactions, for instance in free-ranging dogs or stable groups of dogs hosted in day-care facilities.

## Supplemental Information

10.7717/peerj.20609/supp-1 Supplemental Information 1 Codebook for raw data.

10.7717/peerj.20609/supp-2 Supplemental Information 2 Supplemental information.

10.7717/peerj.20609/supp-3 Supplemental Information 3 ARRIVE checklist.

10.7717/peerj.20609/supp-4 Supplemental Information 4 Example of dog dyad interacting with the apparatus. In first part of the video, both dogs coordinating on hare and obtaining low value reward. In second part, both dogs coordinating on stag and obtaining high value reward.

## Additional Information and Declarations

### Data Availability

The following information was supplied regarding data availability:

The raw data, code, and a video are available at OSF: Mendes, Juliana W. W. 2025. “Dog_dog_cooperation.” OSF. November 25. doi: 10.17605/OSF.IO/25WAM.
