# Supplementary material for: Pet dogs prefer to work alone than to engage in a challenging cooperative task with conspecifics
Source: PeerJ. 2026 Jan 27;14:e20609. doi: 10.7717/peerj.20609 (PMC12857557; doi:10.7717/peerj.20609)
Supplement: Supplemental Information 2 [file peerj-14-20609-s002.docx]

**Supplemental information**

**Dog training**

Dogs were trained on a separate apparatus that had the same mechanics as the experimental apparatus but looked different and was stationary. This approach ensured minimal training in regard to the actual experimental set-up while giving the dogs the knowledge on how perform the action to obtain a reward. The food used for the training was different than the LVR and HVR used in the test (i.e.: we used cheese for the training for dogs that had sausage as the HVR, and sausage for dogs that had cheese as the HVR; see below for more details regarding the food used during testing).

One dog of the dyad was trained to pull on the drawer, and the other dog to pull on the rope. The owners were asked if the dogs had a preference for using the paw or the mouth, and the decision was made based on the answer as to facilitate training. If neither dog had a preference, the choice was randomized.

For the training with the drawer, while the dog was close to the apparatus, the experimenter showed them food and put it on the plate. Then, whenever the dog touched the handle with the paw, they received a piece of food. After five trials in which the dog only needed to touch the handle, the experimenter waited until the dog made any pulling motion before rewarding it (e.g. shaping) (Lindsay, 2013). This process continued until the dog completely pulled the drawer out and ate the food from the plate. For the dogs that were trained on the rope-pulling apparatus, while the dog was close to the apparatus, the experimenter showed him or her food and put it on the plate. Then, when the dog grabbed the rope with its mouth, he or she received a piece of food. After five trials in which the dog only needed to grab the rope, the experimenter waited until the dog pulled it slightly before rewarding it (e.g. shaping). This process continued until the dog pulled the plate completely out and ate the food. In both cases, the dog was considered trained when they performed the action and ate the food 10 times in a row without encouragement of the trainer. The owner was present in the room where the training was taking place, eventually encouraging the dog to look for the food when the dog was not interacting with the apparatus. The second dog was also in the room, held by the owner.

**
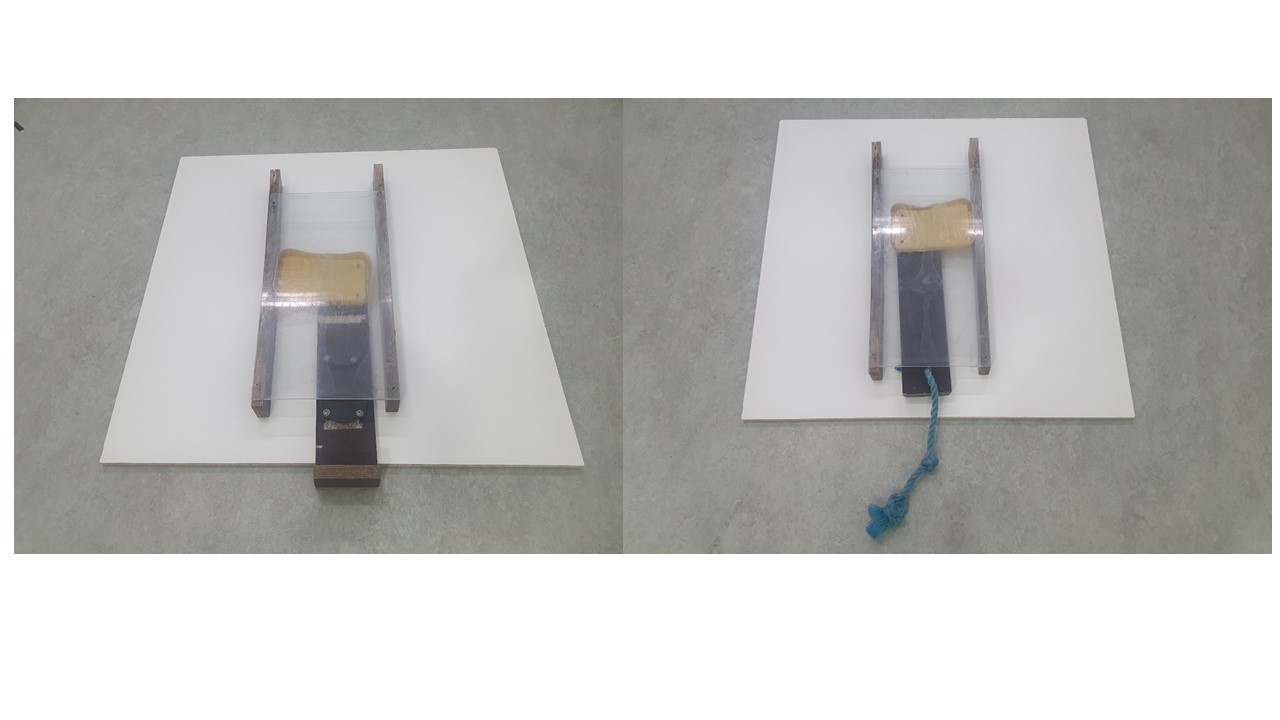
**

SI Fig. 1: Pictures of the training apparatuses. A drawer that dogs could pull with the paw and obtain food from the yellow container (left) and a rope that dogs could pull with the mouth and obtain food from the yellow container (right).

**Food preference test**

As dogs have problems with quantity discrimination (Range et. al, 2014), we used quality differences for the outcomes of the stag hunt game. We experimentally tested for individual food preferences using a food preference test (Brucks et al., 2016). The dog started sitting next to the owner while the experimenter baited a plate with one piece of kibble with her back turned to the dog. The experimenter showed the plate to the dog and allowed it to smell it before placing the plate on the floor 60cm away from the dog. The experimenter then said “okay” and allowed the dog to approach. If the dog ate the food, the experimenter picked up the plate and baited it again. If the dog ate the food six times in a row, this was established as a palatable LVR If the dog did not eat the kibble, a new food would be tested. However, all dogs ate the kibble.

For the second part, the dog sat by the owner again while the experimenter baited one plate with kibble and the other with a food the owner informed was preferred by the dog. The options were sausage, cheese, or commercial treats brought by the owner. The experimenter showed both plates to the dog and allowed it to smell them before placing them on the floor 60cm away from the dog and 60cm cm apart from each other. The experimenter then said “okay” and allowed the dog to move freely. After the dog ate one food, the other was immediately removed. This was repeated 12 times, alternating the position of each food (right of left) between trials. When the dog chose the new food at least nine times, that food was considered the HVR. If the dog chose the new food eight times or less, we repeated the procedure with a different type of food (details in the supplementary material). If the dog never formed a preference, they were included in the control group.

The same procedure was repeated for the second dogs. The first attempt was to keep the LVR and HVR consistent with the first dog. If the second dog did not form the same preference, however, it was possible for dogs to have different rewards.

SI Table 1. Participants and their characteristics.

| **Dog 1** | | | | **Dog 2** | | | | |
| --- | --- | --- | --- | --- | --- | --- | --- | --- |
| **Name** | **Breed** | **Age** | **Sex** | **Name** | **Breed** | **Age** | **Sex** | **Group** |
| Lieserl | Border collie | 1 | Female | alma | Border collie | 1 | Female | Experimental |
| Aquila | Border collie | 9 | Female | nox | Border collie | 1 | Male | Experimental |
| Popeye | American staffordshire terrier | 10 | Male | lili | Mixed breed | 1 | Female | Experimental |
| Emma | German shepherd | 9 | Female | vegas | German shepherd | 2 | Female | Experimental |
| Floki | Australian shepherd | 1 | Male | mexx | Australian shepherd | 11 | Male | Experimental |
| Raya | Mixed breed | 1 | Female | knodel | Mixed breed | 1 | Male | Experimental |
| Diego | Border terrier | 6 | Male | masha | Mixed breed | 3 | Female | Experimental |
| Chivas | Siberian husky | 8 | Male | glenn | Siberian husky | 6 | Male | Experimental |
| Lina | Mixed breed | 7 | Female | lola | Mixed breed | 2 | Female | Experimental |
| Roxy | Irish setter | 8 | Female | ava | Irish setter | 4 | Female | Experimental |
| Ilvi | Smooth collie | 10 | Female | mona | Smooth collie | 9 | Female | Experimental |
| Akido | Mixed breed | 7 | Male | lexi | Mixed breed | 3 | Female | Control |
| Junior | American pitbull terrier | 7 | Male | lilith | Labrador retriever | 6 | Female | Control |
| Nikita | Australian shepherd | 4 | Female | jack | Australian shepherd | 1 | Male | Control |
| Murphy | Mixed breed | 3 | Male | mephisto | Border collie | 7 | Male | Control |
| Simba | Border collie | 9 | Male | nala | Border collie | 7 | Female | Control |
| Bonnie | Labrador retriever | 5 | Female | ara | Labrador retriever | 7 | Female | Control |
| Riley | Border collie | 5 | Female | dazzle | Border collie | 1 | Female | Control |
| Angus | Border terrier | 4 | Male | brexit | Border terrier | 3 | Male | Control |
| Finni | Mixed breed | 7 | Male | ganesh | Mixed breed | 6 | Male | Control |
| Kukie | Border terrier | 7 | Female | madlain | Border terrier | 7 | Female | Control |
| Pia | Whippet | 7 | Female | suri | Belgian shepherd malinois | 12 | Female | Control |
| Bonnie | Labrador retriever | 2 | Female | nugget | Labrador retriever | 4 | Female | Control |
